# Management of two cases of tracheobronchial management of two cases of tracheobronchial papillomatosis at tertiary hospital in Indonesia: A case report

**DOI:** 10.1016/j.ijscr.2021.106054

**Published:** 2021-05-29

**Authors:** Rizka Fathoni Perdana

**Affiliations:** Department of Otorhinolaryngology, Head and Neck Surgery, Faculty of Medicine, Universitas Airlangga/Dr. Soetomo General Hospital, Indonesia

**Keywords:** Recurrent respiratory papillomatosis, Tracheobronchial papillomatosis, Aggresive papillomatosis, Bronchoscopy, Juvenile papillomatosis, Case report

## Abstract

**Introduction and importance:**

Tracheobronchial papillomatosis is an aggressive form of RRP with the spread of papillomas to the subglottis, trachea, bronchus and pulmonary parehchyma. Surgical operation for removing the papilloma is extremely difficult and need a lot of periodical bronchoscopy.

**Case presentation:**

The first patient was a 25-year-old male who had an RRP history since the age of 6 months. Patients undergo papilloma cleaning surgery every 2 to 4 months. So far, the patient has undergone 88 operations. The frequency of surgery did not decrease even though the patient had reached adulthood. Moreover, the second patient was a 9-year-old woman suffering from RRP since the age of 6 months. The history of surgery has been carried out four times. The patient did not regularly go to the hospital. Consequently, the papilloma blocked the airway and the patient underwent tracheotomy at 3 years-old. A recent endoscopic examination showed papillomas growing in the trachea so that the tracheal stoma was maintained at this time to keep the upper airway patent and access for surgery.

**Clinical discussion:**

Endoscopic removal surgery is required for larynx and tracheobronchial papillomas. Debulking through bronchoscopy regularly in order to maintain the airway patency. Tracheal stoma is needed for surgical access. Hence, accurate monitoring of disease progression and potential changes in malignancy is needed.

**Conclussion:**

Tracheobronchial papillomatosis is very rare disease that needs periodically surgery for clean the tumor and monitoring the possibility for malignancy.

## Highlights

• Recurrent respiratory papillomatosis (RRP)
• Human Papillomavirus (HPV); type 6 and 11
• Tracheobronchial system and lung parenchyma
• Endoscopic surgery is required on the larynx-trachea.
• Monitoring of disease progression and potential changes to malignancy is needed.

## 1 Introduction

Recurrent respiratory papillomatosis (RRP) is a disease caused by Human Papillomavirus (HPV), especially low-risk HPV with manifestations of papilloma growth in the respiratory tract. The types of HPV that are heavily involved are types 6 and 11 [1]. The nature of this disease is the growth of recurrent exophytic lesions. Although RRP is a benign tumor it has morbidity and mortality consequences because of papilloma growth that often causes obstruction of the upper airway [2]. This could be applied to an aggressive disease as well, it can spread to the lower respiratory tract and lungs that make a poor prognosis [3].

The true incidence and prevalence of RRP are unknown. However, most of it is due to the time delay between the onset of voice changes or hoarseness with a definitive diagnosis [4]. Reports on RRP incidents in the Danish population was about 3.84 cases per 100.000 (children were about 3.62 per 100.000 and adults around 3.94 per 100.000) [5]. Whereas in the United States, the estimated incidence in the pediatric population was 4.3 per 100.000 children and 1.8 per 100.000 adults [6].

RRP can affect humans of all ages, it has been identified that the youngest ever reported was a year old and the oldest age was 84 years. There were two onsets; RRP onset of childhood (childhood-onset RRP) and juvenile-onset RRP (JORRP) with definition the child's age is under 12 years for the first diagnosed. The most common age range in RRP types of children is at ages 2 to 4 years. Adult-type RRP (adult-onset RRP/AORRP) has a peak age between 20 and 40 years and males are slightly more common. The distribution of males and females is balanced and there is no difference between the frequency of surgery between sexes or ethnicities. Juvenile type RRP is more common and more aggressive than adult type [1].

Patients can visit the hospital with various symptoms and physical examination findings due to the highly varied RRP aggressiveness. As well as the varied number of respiratory tract locations involved. Subtle changes in voice over a long period of time may not be noticed by adult patients, because it may never too deep into a hoarse voice, dysphonia, or tightness [7]. There are two types of RRP based on the natural course of the disease, namely aggressive and non-aggressive. An aggressive type RRP is defined based on a history of tumor cleansing operations that happened more than 10 times, three or more history of surgery in a one-year period, and papilloma involvement in the subglottis, trachea or bronchi. The primary location for papilloma growth is in the laryngeal area but can be extended to the lower airway [8].

Tracheobronchial papillomatosis is a form of aggressive RPP with the spread of papillomas to the subglottis, trachea, bronchi and to the lung parenchyma areas [9]. The spread of papillomas in the bronchial epithelium is part of HPV infection. The incidence of tracheobronchial papillomatosis was range about 43 cases per 1 million for children-types of RRPs and 18 cases per 1 million for adult. Male suffer 4 times more than female. TBP is rarely a disease that stands alone because it is often a continuation of a laryngeal papilloma [10].

Two cases of child-type RRP was reported with papilloma extension to the tracheobronchial and even lung parenchyma. Both patients were diagnosed with RRP since age six months old. The course of both diseases are includes as an aggressive type of RRP so that tracheotomy is still maintained untill today. Both patients underwent microscopic laryngeal surgery 88 times and 4 times. One patient died of respiratory failure and one still survives until now.

## 2 Case presentation

The first patient was a man aged 25 years who has a history of RRP since he was 6 months old. Patients' first visit was complaints of shortness of breath. The complaint was preceded by a hoarse voice since 5 months ago. Endoscopic examination of the larynx showed a mass of laryngeal papillomas that filled the larynx so that the airway became narrow. Patients diagnosed with pediatric laryngeal papilloma type and upper airway obstruction. Patients undergo tracheotomy and proceed with microscopic laryngeal surgery to clear the papilloma mass.

Patients undergo papilloma cleansing surgery every 2 to 4 months. The patients who live outside the city and far from the health center that capable of performing laryngeal surgery cause patients to repeatedly experience upper airway obstruction so that they undergo a repeated tracheotomy. It was obtained a history by one of the patient that has undergone tracheotomy 4 times due to repeated upper airway obstruction. Until the age of 25 years, patients have undergone surgery 88 times already. The frequency of surgery does not decrease even though the patient has reached adulthood. Papilloma of the trachea began to spread to the bronchi since the age of 12 years so that the tracheocele was maintained from 1992 until now.

**Fig. 1:**
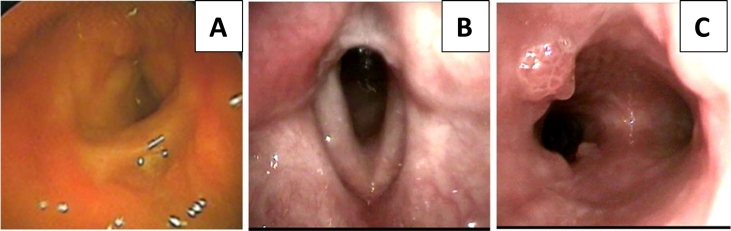
A: laryngoscopic examination in the first patient after 87th times microscopic laryngeal surgery, stenosis of the larynx appears, B: second patient after the last operation, laryngeal clear of papilloma mass C: first patient 5 months after the last operation, visible mass minimal papilloma in the trachea and left main bronchi.

**Fig. 2:**
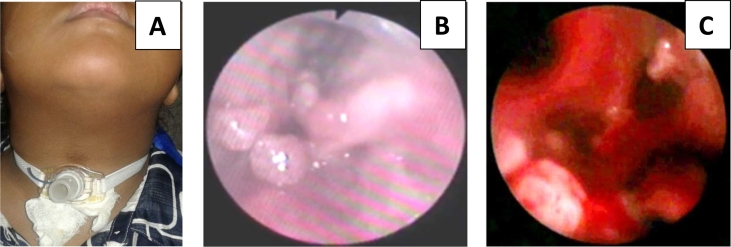
A: Second patient with tracheotomy. B: the first laryngoscopy examination shows papilloma growth in the larynx which causes airway obstruction. C: papilloma growth in the tracheal lumen to bronchial branching.

Repeated surgery causes stenosis in the larynx (Fig. 1A) so that the tracheocele is maintained. Another benefit of maintaining tracheoculosis is that it facilitates bronchoscopy when cleaning papillomas in the trachea-bronchi. A final endoscopic examination shows papilloma implantation in the trachea to the proximal right main bronchi (Fig. 1C). HPV virus examination by the PCR method showed that the presence of HPV type 11 infection in patients with papilloma preparations (Fig. 2).

**Fig. 3:**
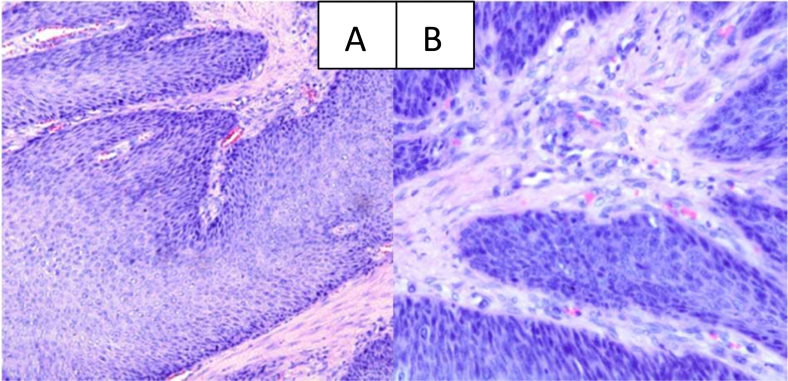
A and B. Histopathological features of the tissue of the first patient's papilloma specimen. The papilloma squamous epithelium is covered with papilloma with moderate dysplasia. It also appears that the epithelium has poikilocytosis and fibrous tissue in the stroma.

**Fig. 4:**
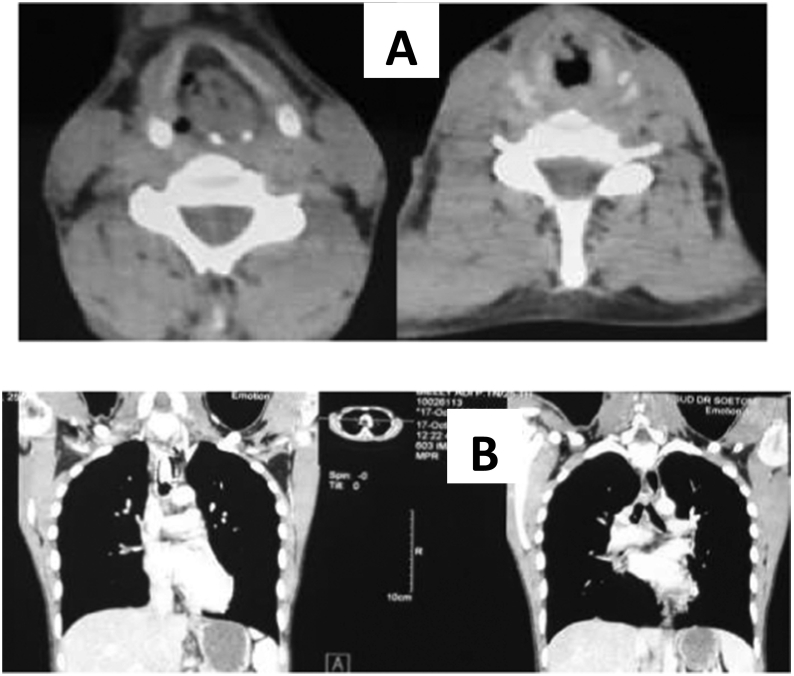
A: axial cuts in the neck show mass that fills the larynx, causing almost complete obstruction. B: A thoracic cut. Tracheocanul fills the trachea to carina. The extensive disease is indicated by multiple nodules in the right lung and the medial lobe of the left lung.

Histopathological examination of papilloma tissue was revealed the squamous epithelium coated with papilloma with moderate dysplasia and epithelium that appeared to be koilocytosis (Fig. 3). CT-scan of the cervical-thoracic thoracic (Fig. 4) shows laryngeal narrowing due to papilloma mass and in the thorax papilloma growths are found in the trachea to the carina and nodules are found in the right lung parenchyma.

The second patient was a female aged 9 years old who has suffered from RRP since she was 6 months old. A history of surgery that has been undertaken was four times. Patients do not control on time so that the papilloma mass fills the larynx and becomes short of breath. At the age of 3, the patient has a tracheotomy. A final endoscopic examination showed papillomas growing on the trachea so that the tracheal cannula was maintained to date to keep the upper airway patent (Fig. 1B).

## 3 Discussion

Many RRP sufferers come to our hospital, in fact, we have the most patient visits in Indonesia. It was reported by Perdana (2020) that 4 out of 34 RRP patients had papilloma implantation in the distal, including the trachea to the bronchi. We chose 2 cases with complete documentation and data. Both cases were classified as juvenile-type RRP because papillomas suffered from 6 months of age. The primary location of RRP is the larynx with early complaints of hoarseness [6,11]. Early detected for papillomas often appear in the area of transformation in the larynx, namely the change of squamous epithelium into ciliated columnar epithelium in the upper respiratory tract [12]. However in this study, hoarseness complaints are not a strong reason for patients to visit the doctor, so they were often postponed. Papillomas will grow to fill the larynx over time if it not immediately being handled. The airway will become narrowly closed by the mass so the patients will complain of shortness of breath. Stridor and upper airway obstruction are the main complaints of patients going to the hospital, not hoarseness. This is caused by the time lag between the onset of the disease and the diagnosis [11]. Patients who visit the hospital in the condition of experiencing airway obstruction will require tracheotomy in an effort to maintain airway patency. Papilloma tissue cleansing surgery will be performed after the obstruction is resolved. The purpose of the cleaning operation is an effort to clean the larynx. The goal of microscopic laryngeal surgery is to keep the upper airway free and improve sound quality of voice [14].

The first patient has undergone surgery 88 times and operating frequency in a year are ranges from 2 to 4 times. The frequency of surgery does not decrease even though the patient has been grown as teenager. In addition, because of the patient undergone so many operations it cause complications in the larynx in the form of stenosis. The stenosis makes the patient have to breathe through the canule. The clinical of both patients is aggressive. This is in accordance with past studies The journey of both patients is aggressive. This is in accordance with previous research that most juvenile papillomas will experience an aggressive course of disease. An indicator of aggressive disease is rapid papilloma mass recurrence that requires surgery more than 3 times in one year. In addition, the patients of this study subject meet the above criterias [16].

Another sign of aggressiveness is distal papilloma implantation [17]. Both patients suffer papillomatosis in the trachea, bronchi and lung parenchym. Both patients who underwent emergency tracheotomy surgery were caused by a lack of discipline in control regularly so that upper airway obstruction could not be avoided. Tracheotomy is also an aggressive form of the disease [17]. Tracheocanule must be removed immediately because it has the potential to cause more distal papilloma growth. However, due to geographical reasons, the location of the patient's house is far from the health center and the low level of awareness of the patient to go to the hospital with discipline causes makes the doctor to delay the release of the tracheotomy. Tracheotomy in RRP is still controversial because it is thought to facilitate the spread of papillomas into the distal airway.^19^ This is caused by injury to the stoma making epithelialization changes. Deeper distribution opens a chance for papillomas to spread to the trachea-bronchi then to the lung parenchyma. This causes the two patients still can not be removed due to implantation of papilloma far distal papilloma, namely to the trachea to the main bronchi.

Tracheacanule in both patients is still maintained until now. It aims to maintain the airway because papillomas in both patients have a very rapid growth. In addition, the spread of papillomas to the trachea and bronchi requires a complex operative actions that are more easily done through the stoma that is maintained. In the first case, the patient has identified the HPV type-11 as the cause. Allegedly, HPV type 11 causes more aggressive disease compared to type 6. RRP aggressive type in our hospital dominates with a percentage of 80% of all RRP incidents. This is reported that 100% of RRP patients have HPV infection in papilloma tissues with dominated by HPV type 6 [15]. Tracheobronchial papillomatosis is an aggressive form of RRP. So, it is very difficult to treat and requires repeated endoscopic action to reduce papilloma mass. A surgical approach is a complicated thing and must be done repeatedly. Both patients are require periodic papilloma extraction through bronchoscopy to keep the airway free. The most common recurring surgery complication is airway stenosis, both laryngeal and tracheal stenosis. Complications in the form of laryngeal stenosis occur in the first case. Repeated endolaryngeal surgery causes the spread of papillomas to the lower airway even to the lung parenchyma. Another hand, to repeated surgeries to clear papilloma masses, close and continuous monitoring of progressive pulmonary organ progression and the possibility of transformation into malignancy is possible. In addition, all reports that we have done following the SCARE 2020 Guideline: Updating Consensus Surgical CAse REport (SCARE) Guidelines, International Journal of Surgery 2020 and the case report ethical protocol from Soetomo Teaching Hospital, Surabaya [18].

## 4 Conclusion

Two cases of aggressive type RRP have been reported to have spread to the tracheobronchial system and involve the lung parenchyma. Endoscopic surgery is required on the larynx-trachea through bronchoscopy also maintaining tracheocele to keep the airway patency. Then, significant monitoring of disease progression and potential changes to malignancy is needed.

## Source of funding

The research was funded by the authors.

## Ethical approval

Taken from Ethical Committee of Dr. Soetomo General Hospital, Surabaya Indonesia.

## Consent

Written informed consent was obtained from the patients for publication of this case report and accompanying images. A copy of the written consent is available for review by the Editor-in-Chief of this journal on request.

## Registration of research studies

Not applicable.

## Guarantor

Rizka Fathoni Perdana.

## Provenance and peer review

Not commissioned, externally peer-reviewed.

## CRediT authorship contribution statement

All research activities have been done by the author (Rizka Fathoni Perdana as the single author), including data collection, writing, drafting, review, and editing.

## Declaration of competing interest

The authors report no declarations of interest.

## References

[1] Larson D.A., Derkay C.S. (2010). Epidemiology of recurrent respiratory papillomatosis. Apmis..

[2] Lee J.H., Smith R.J. (2005). Recurrent respiratory papillomatosis: pathogenesis to treatment. Curr. Opin. Otolaryngol. Head Neck Surg..

[3] Goon P., Sonnex C., Jani P., Stanley M., Sudhoff H. (2008). Recurrent respiratory papillomatosis: an overview of current thinking and treatment. Eur. Arch. Otorhinolaryngol..

[4] Doyle D.J., Gianoli G.J., Espinola T., Miller R.H. (1994). Recurrent respiratory papillomatosis: juvenile versus adult forms. Laryngoscope..

[5] Lindeberg H. (1990). Laryngeal papillomas: the epidemiology in a Danish subpopulation 1965-1984. Clin. Otolaryngol..

[6] Derkay C.S., Wiatrak B. (2008). Recurrent respiratory papillomatosis: a review. Laryngoscope..

[7] Andrus J.G., Shapshay S.M. (2006). Contemporary management of laryngeal papilloma in adults and children. Otolaryngol. Clin. N. Am..

[8] Buchinsky F.J., Donfack J., Derkay C.S., Choi S.S., Conley S.F., Myer C.M. (2008). Age of child, more than HPV type, is associated with clinical course in recurrent respiratory papillomatosis. PLoS One.

[9] Lam F.C.Y., Tsang T.K., Fung H.S., Wai M.W., Kwan T.L., Chan S.C.H. (2004). Laryngeal papillomatosis with pulmonary spread: case report and review. J HK Coll. Radiol..

[10] Harris K., Chalhoub M. (2011). Tracheal papillomatosis: what do we know so far? Chron Respir Dis.

[11] Perdana R.F., Herawati S., Surarso B., Aksono E.B. (2020). Correlation of aggressivity papilloma recurrent respiratory tract with human papillomavirus types 6 and 11. Med. Leg. Updat..

[12] McKaig R.G., Baric R.S., Olshan A.F. (1998). Human papillomavirus and head and neck cancer: epidemiology and molecular biology. Head Neck J. Sci. Spec. Head Neck..

[14] Perdana R.F., Herawati S., Yusuf M., Kristyono I., Nugroho P.S. (2020). Correlation between P53 and KI67 with aggressiveness factor in recurrent respiratory papillomatosis. Syst. Rev. Pharm..

[15] Wiatrak B.J., Wiatrak D.W., Broker T.R., Lewis L. (2004). Recurrent respiratory papillomatosis: a longitudinal study comparing severity associated with human papilloma viral types 6 and 11 and other risk factors in a large pediatric population. Laryngoscope..

[16] Donne A.J., Hampson L., Homer J.J., Hampson I.N. (2010). The role of HPV type in recurrent respiratory papillomatosis. Int. J. Pediatr. Otorhinolaryngol..

[17] Blackledge F.A., Anand V.K. (2000). Tracheobronchial extension of recurrent respiratory papillomatosis. Ann. Otol. Rhinol. Laryngol..

[18] Agha R.A., Franchi T., Sohrabi C., Mathew G., for the SCARE Group (2020). The SCARE 2020 guideline: updating consensus surgical CAse REport (SCARE) guidelines. Int. J. Surg..

